# The complete mitochondrial genome and phylogenetic analysis of *Neorhodomela munita*

**DOI:** 10.1080/23802359.2021.1966343

**Published:** 2021-08-25

**Authors:** Zilin Jiang, Ruoran Li, Yutong Cui, Xuli Jia, Tao Liu, Xumin Wang, Jiangyong Qu

**Affiliations:** a College of Life Sciences, Yantai University, Yantai, PR China; b Laboratory of Genetics and Breeding of Marine Organism, College of Marine Life Sciences, Ocean University of China, Qingdao, PR China

**Keywords:** *Neorhodomela munita*, mitochondrial genome, phylogenetic analysis

## Abstract

*Neorhodomela munita* (Perestenko) Masuda 1982 is distributed in the coastal areas of Shandong and Liaoning in China, and also in Japan. In this study, the complete nucleotide sequence of the circular mitochondrial DNA of the red alga *Neorhodomela munita* has been determined. The complete mitochondrial DNA sequence of *Neorhodomela munita* was 25,318 bp in length with an overall GC content of 25.1% and encoded 23 protein-coding genes, two ribosomal RNAs and 24 transfer RNAs. Phylogenetic tree showed that *Neorhodomela munita* clustered together with *Choreocolax polysiphoniae*. The phylogenetic analysis may provide a better understanding of the evolution of the Rhodophyta species.

*Neorhodomela munita* (Perestenko) Masuda 1982 belongs to *Neorhodomela* (Rhodomelaceae; Ceramiales; Florideophyceae; Rhodophyta). It is distributed in the coastal areas of Shandong and Liaoning in China, and also in Japan (Bangmei 2011). It mainly lives in swamps of intertidal zone and rocks, and plays an important role in the ecological environment of intertidal zone. As a unique group of organisms evolving from prokaryotes and eukaryotes, algae are the key to decipher the origin and evolution of eukaryotes. As the product of the first endosymbiosis, mitochondrial genomes can provide nucleotide or amino acid sequence data for phylogenetic analysis, and many structural characteristics (such as genome size, GC content, gene content, and genome collinearity) can directly provide evidence for system development (Martin and Russell 2003; Robba et al. 2006). In this study, we sequenced, assembled, and annotated the mitochondrial DNA of *Neorhodomela munita*, conducted a systematic genome study to reveal the evolutionary of its mitochondrial DNA, and studied the phylogenetic relationships with several other algae.

*Neorhodomela munita* was collected from Badaguan Area, Qingdao, Shandong Province, China (36°02′57′′N, 120°20′52′′E), and stored in −80 °C refrigerator at the Culture Collection of Seaweed at the Ocean University of China (Tao Liu, liutao@ouc.edu.cn) under the voucher number 2016040001. The determination of the complete *Neorhodomela munita* mitochondrial genome sequence was conducted by next-generation sequencing methods. Total DNA was extracted by using the modified CTAB method (Doyle and Doyle 1990). Paired-end reads were sequenced with the use of Illumina HiSeq Ten system (Illumina, San Diego, CA). The experimental methods and data analysis were followed by the previous reports (Tamura et al. 2013; Liu et al. 2019).

The complete mitochondrial genome of *Neorhodomela munita* is a circular DNA molecule with the length of 25,318 bp (GenBank accession number: MW750196). Overall, GC content of the complete mitochondrial genome was 25.1%. The mitogenome contained 49 genes (23 protein-coding genes, two *rRNAs*, and 24 *tRNAs*). The nucleotide consisted of 39.7% A, 12.9% G, 35.3% T, and 12.2% C. The length of coding region accounted for 91.44% of the total length. All the 23 protein-coding genes of *Neorhodomela munita* use ATG as the start codon. Twenty of 23 (86.96%) protein-coding genes (*nad*4L, *cox*2, *cox*3, *atp*4, *cob*, *nad*6, *sdh*B, *sdh*C, *atp*9, *rps*11, *nad*3, *nad*1, *nad*2, *sdh*D, *nad*4, *nad*5, *atp*8, *atp*6, *tat*C, and *rps*12) end with the TAA stop codon, and three of 23 (13.04%) protein-coding genes (*rps*3, *rpl*16, and *cox*1) end with TAG.

To construct the concatenated alignment of mitochondrial genes, conserved 12 protein-coding genes (*atp*6, *cox*1, *cox*2, *cox*3, *cyt*b, *nad*1, *nad*2, *nad*3, *nad*4, *nad*4L, *nad*5, and *na*d6) from 26 red algae and two green algae including *Neorhodomela munita* were used, and the gene sets were aligned by MrBayes version 3.1.2 software, Uppsala, Sweden (Ronquist and Huelsenbeck 2003). The phylogenetic analysis was run until the average standard deviation of split frequencies was below 0.01. *Auxenochlorella protothecoides* (NC_026009.1) and *Ulva prolifera* (NC_028538.1) served as the out-group. Poorly aligned regions were removed by using the Gblocks server (Castresana 2000). Phylogenetic tree showed that all red algal taxa were clearly separated according to their original class (Figure 1). In phylogenetic tree, *Neorhodomela munita* has the closest relationship with *Choreocolax polysiphoniae*, followed by the *Vertebrata lanosa* and *Dasya binghamiae* in order Ceramiales. At class Rhodophyta level, Ceramiales, the order of *Neorhodomela munita*, is more closely related to Bonnemaisoniales. This complete mitochondrial genome analysis of *Neorhodomela munita* will help us better understand the evolutionary process of Rhodophyta species.

**Figure 1.**
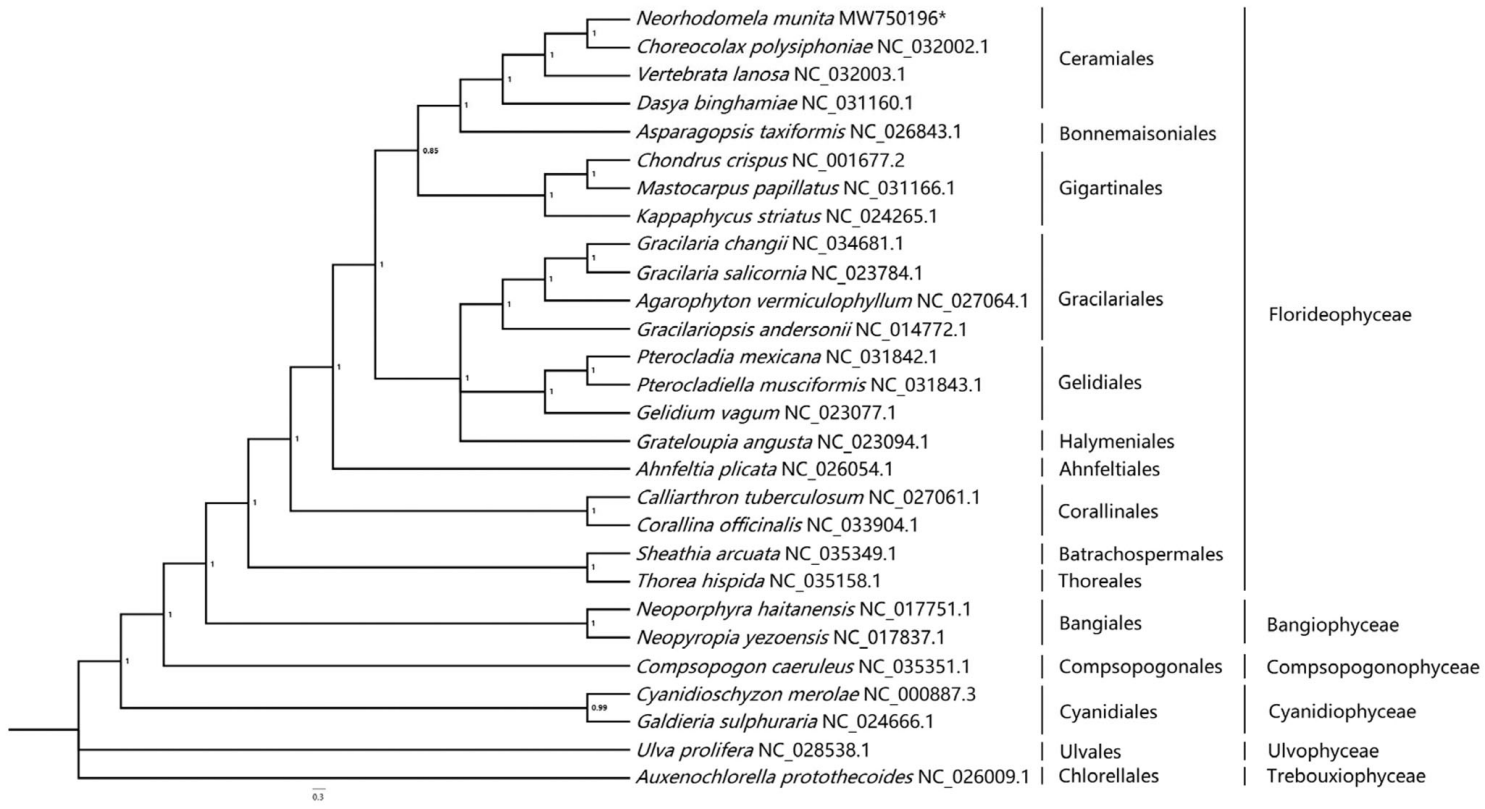
Phylogenetic tree (Bayesian method) based on the complete mitochondrial genomes sequence. Support values for each node were calculated from Bayesian posterior probability (BPP). Asterisks following species names indicate newly determined mitochondrial genomes.

## Data Availability

The genome sequence data that support the findings of this study are openly available in GenBank of NCBI at [https://www.ncbi.nlm.nih.gov] (https://www.ncbi.nlm.nih.gov/) under the accession no.MW750196.
